# Transcranial random noise stimulation (tRNS) improves hot and cold executive functions in children with attention deficit-hyperactivity disorder (ADHD)

**DOI:** 10.1038/s41598-024-57920-3

**Published:** 2024-03-31

**Authors:** Vahid Nejati, Mahshid Dehghan, Shahriar Shahidi, Reza Estaji, Michael A. Nitsche

**Affiliations:** 1 Department of Psychology, Shahid Beheshti University Tehran, P.O. Box: 1983969411, Tehran, Iran; 2 Department of Psychology and Neurosciences, Leibniz Research Centre for Working Environment and Human Factors, Dortmund, Germany; 3 Department of Neurology, University Medical Hospital Bergmannsheil, Bochum, Germany

**Keywords:** Attention deficit-hyperactivity disorder (ADHD), Hot and cold executive functions ventromedial prefrontal cortex (vmPFC), Dorsolateral prefrontal cortex (dlPFC) transcranial random noise stimulation (tRNS), Randomized controlled trials, ADHD

## Abstract

Children with attention deficit-hyperactivity disorder (ADHD) have impaired hot and cold executive functions, which is thought to be related to impaired ventromedial and dorsolateral prefrontal cortex (vmPFC and dlPFC) functions. The present study aimed to assess the impact concurrent stimulation of dlPFC and vmPFC through transcranial random noise stimulation (tRNS), a non-invasive brain stimulation tool which enhances cortical excitability via application of alternating sinusoidal currents with random frequencies and amplitudes over the respective target regions on hot and cold executive functions. Eighteen children with ADHD received real and sham tRNS over the left dlPFC and the right vmPFC in two sessions with one week interval. The participants performed Circle Tracing, Go/No-Go, Wisconsin Card Sorting, and Balloon Analogue Risk Tasks during stimulation in each session. The results showed improved ongoing inhibition, prepotent inhibition, working memory, and decision making, but not set-shifting performance, during real, as compared to sham stimulation. This indicates that simultaneous stimulation of the dlPFC and the vmPFC improves hot and cold executive functions in children with ADHD.

## Introduction

Attention deficit-hyperactivity disorder (ADHD) is a neurodevelopmental disorder characterized by impaired attention and hyperactivity-impulsivity^1^. It is associated with impaired perceptual^2^, attentional^2,3^, temporal^4^, emotional^5^, executive^6^, social^7^, and motivational^8^ processing. Two primary concepts have been proposed to describe abnormal cognitive processing observed in ADHD: the executive^6^ and motivational theories^9,10^. The executive theory assumes that ADHD is characterized by impaired executive functions, while the motivational theory suggests that delay aversion and abnormal reward processing are key factors in ADHD symptoms. The former applies to impaired cold executive functions such as working memory, inhibitory control, and cognitive flexibility, whereas the latter considers temporal, emotional, and social components, and focuses on impaired hot executive functions, such as risky decision making and delay discounting in individuals with ADHD. Cold executive functions encompass cognitive processes characterized by analytical and rule-based reasoning, emphasizing objectivity and logic. In contrast, hot executive functions integrate emotional and motivational factors into cognitive processes^11^.

At the neural level, impaired functionality of the dorsolateral prefrontal cortex (dlPFC) and ventromedial prefrontal cortex (vmPFC), two distinct prefrontal areas supporting cold and hot executive functions, respectively are involved in these cognitive deficits^12^.

In healthy individuals, physiological evidence partially supports this differentiation. Executive functions are associated with the dlPFC^13,14^, while motivational processing is linked to the vmPFC^15^. An earlier neuroimaging study found that the activity of these two structures is anticorrelated and mutually exclusive during task performance in healthy adults^16^. This differentiation holds however not for all studies in the field. Several studies found a role of the dlPFC in emotional processing^17–20^, and an involvement of the vmPFC in executive processing, including working memory^21^, cognitive flexibility^22^, and inhibitory control^22^.

In ADHD, beyond these two basic executive and motivational theories, the dual pathway theory attributes ADHD symptoms to both, executive and motivational impairment. This theory presumes that ADHD involves a dysregulated cognitive control pathway, including the dlPFC and dorsal striatum, and an impaired reward pathway, including the vmPFC, ventral striatum, amygdala, and anterior cingulate cortex^23,24^.

Non-invasive brain stimulation (NIBS) provides an opportunity to modulate the pathophysiological underpinnings of psychopathologic conditions through the alteration of neural excitability^25,26^. NIBS includes two main techniques: transcranial magnetic stimulation (TMS) and transcranial electrical stimulation (tES). TMS induces a magnetic field in the specific target regions of the brain, resulting in suprathreshold depolarization of the cortical neurons within the targeted area^27^. tES alters cortical excitability through the application of an electrical current over the brain, and involves three main available methods: transcranial direct current stimulation (tDCS), transcranial alternating current stimulation (tACS), and transcranial random noise stimulation (tRNS). tDCS alters neuronal resting membrane potentials, and enhances, or reduces cortical excitability under anodal and cathodal electrodes, respectively^26,28^. tACS applies an alternating current over the scalp to entrain, or synchronize cortical oscillations^29^. tRNS delivers a random balanced electrical oscillation spectrum to the brain. Higher frequencies, ranging from 100 to 640 Hz, enhance excitability of the underlying cerebral regions^30^. Although the exact underlying mechanisms of action of tRNS are not yet fully understood, several concepts have been developed. First, opening of Na + channels has been proposed^31^. Second, increased synaptic plasticity through stochastic resonance, based on an addition of noise to targeted cortical neural networks might increase the sensitivity of the system to weak signals^32^. Third, tRNS might modulate the balance between excitatory and inhibitory neuronal activity in the brain^31^.

The majority of tES studies in ADHD so far used tDCS for modulation of the dlPFC and vmPFC to improve hot and cold executive functions. Anodal left dlPFC/cathodal right vmPFC stimulation has been shown to improve working memory^33,34^, and cognitive flexibility^33^. Improvement of hot executive functions, more conservative decision making, and lower discounting rates have been described with a reversed electrode arrangement, namely anodal right vmPFC/cathodal left dlPFC stimulation^17^. This finding shows that upregulation and/or downregulation of these areas improves cold and hot executive functions in children with ADHD. In accordance, anodal left/cathodal right dlPFC stimulation improved working memory performance^33^, while anodal stimulation of the right dlPFC with an extracranial reference electrode improved inhibitory control in children with ADHD^35^. However, an online bilateral anodal left and right dlPFC stimulation protocol with extracranial reference electrodes did not improve working memory, inhibitory control and cognitive flexibility, as compared with sham stimulation in children with ADHD^36^. A face to face tES study in ADHD recently compared tDCS (anodal dlPFC/cathodal vmPFC stimulation) with tRNS (bilateral dlPFC stimulation) in ADHD, and found a larger tRNS effect on ADHD symptoms, and working memory performance^37^. Further research is necessary to draw valid conclusions about the superiority of specific stimulation protocols, considering the inhomogeneous electrode placement used for the tES modalities in this study and the limited number of tRNS studies conducted in ADHD. In summary, previous research has delineated the favorable effects of stimulating the dlPFC and vmPFC on both cold and hot executive functions in individuals with ADHD. Nonetheless, the simultaneous upregulation of these regions through tRNS and its influence on both cold and hot executive functions remains unexplored to date.

The current study targeted impaired hot and cold executive functions in children with ADHD. We assumed the vmPFC and dlPFC as the neural underpinnings of these impairments, and expected an improvement of these functions during real compared to sham tRNS via increasing excitability these areas. Our objective was thus to assess how simultaneous upregulation of the dlPFC and vmPFC affects hot and cold executive functions in children with ADHD. We hypothesized an enhancement in both hot and cold executive functions through the simultaneous targeting of the dlPFC and vmPFC using tRNS.

## Material and methods

### Participants

18 children with ADHD, aged between 7 and 12 years (mean age = 9.89, standard deviation = 1.90; 12 boys and 6 girls), were recruited from the Mofid Children’s Hospital (Tehran, Iran). This age group experiences more pronounced impairments in executive functions compared to adolescents and adults^38^. Sample size was determined with G*power software, which suggested a minimum of 16 participants for a one-way repeated measures ANOVA with a power of 0.95, an alpha level of 0.05, and a medium effect size (f = 0.40). All participants were diagnosed with ADHD, based on the Diagnostic and Statistical Manual of Mental Disorders 5th edition, by a child and adolescent psychiatrist. Out of the 18 participants, 12 were taking medication such as methylphenidate and fluoxetine, which they stopped taking 12 h prior to the study sessions. All participants were right-handed based on their report and had normal or corrected to normal vision. None of the participants had a history of traumatic brain injury, or other neurodevelopmental or psychiatric disorders, based on psychiatric report. The study adhered to the ethical standards outlined in the Declaration of Helsinki from 1975, revised in 2013, and was approved by the national ethical committee (for demographic sample characteristics see Table 1).

**Table 1:** Demographic characteristics and ADHD- rating of participants.

Variables	M (SD)
Demographic characteristics	
Age (Years)	9.89 (1.90)
Education (Years)	3.89 (1.90)
Gender (Male/Female)	12/6
SNAP—rating scale	
Attention deficit symptoms	16.33 (3.32)
Hyperactivity symptoms	15.77 (3.49)
ADHD symptoms	32.11 (6.15)

### Circle tracing task

This task was developed to evaluate ongoing inhibition by exerting inhibitory control during the performance of a motor action^39^. Participants are required to trace a printed circle using their index finger on a card board covered with Plexiglas. The task consists of two trials with different instructions: the first trial involves tracing the circle from the marked start/end point with the individually preferred speed, while the second trial requires participants to trace the circle as slowly as possible, which tests their ongoing inhibitory control. The task has a maximum time limit of 12 min per trial, which is not communicated to the participants. This task has been used in children before^40^, and the outcome measures include the time taken to perform each trial in seconds (TA and TB), and the Circle Tracing Index (CTI), which is calculated as TB minus TA.

### Go/No-Go task

This task was used to evaluate prepotent response inhibition, which involves inhibiting a dominant response when a stop signal is presented^41^. This type of inhibition requires the ability to differentiate between contexts where the target stimulus should be responded to (Go stage) versus ignored (No-Go stage) within the same task. The Go/No-Go and stop signal tasks are commonly used to assess action inhibition. In the current experiment, the task involved a 7 × 7 cm plane appearing at the center of a screen as the Go stimulus, to which the participant had to respond to by pressing the arrow key corresponding to the flight direction of the plane on a keyboard as quickly and accurately as possible. Each trial began with presentation of a fixation cross for 1000 ms, followed by the Go stimulus (remaining on the screen for 1800 ms or until a response was made), and ended with presentation of a second fixation cross (2000 ms minus response time). In 25 out of 100 trials, a beep sound served as a No-Go signal, prompting the participant to withhold the key press. The outcome measures for the task include accuracy and reaction time of the Go stage, and accuracy of the No-Go stage as the main outcome measure.

### 1-back test

This test was applied to evaluate working memory performance^42^. In this test, 100 stimuli, irregular lines, were displayed on a screen in a predetermined sequence. Participants were required to determine whether the sequential stimuli were identical or different by pressing the "same" or "different" buttons. Twenty-five stimuli were similar to the preceding one. The task duration was approximately 7 min, with accuracy and speed serving as performance parameters.

### Wisconsin card sorting task

This task was developed to evaluate cognitive flexibility^43^. Four sample cards are displayed on a screen, which depict different symbols with different colors, and each card depicts different number of symbols. Specifically, these cards feature either one, two, three, or four identical shapes (triangles, stars, crosses, or circles) printed in red, green, yellow, or blue. Beneath the sample cards is a card deck, and participants must use a guessed rule (such as color, number, or shape) to match the top card at the deck with one of the sample cards. They receive accuracy feedback regarding their choices. Once they have made 10 correct matches based on a particular rule as a cluster, the rule changes. If they continue to sort cards based on the previous rule, this is rated as perseveration error. The number of clusters, perseveration errors, and the total time to sort all 64 cards are used as performance measures in this task. Additionally, a speed-accuracy ratio was computed for this task by dividing response time by accuracy.

### Balloon analogue risk task (BART)

BART allows to evaluate risky decision-making abilities^44^. During this task, a virtual balloon is displayed on a computer screen and participants are required to inflate it by pressing a pedal button. Each pump increases the size of the balloon and provides a virtual monetary reward of 1000 RLS in the present study. However, the balloon is programmed to burst after an unpredictable number of pedal presses, with a linear probability function, creating a higher risk of bursting as it becomes larger. If the balloon bursts, any earned money is lost. During a trial, participants can either choose to collect their earnings by pressing the "collect money" button, or continue pumping the balloon to earn more money. Once a non-exploded balloon is collected, the earned money is transferred to a permanent box, and a new balloon appears. The task consists of 30 trials and typically takes around 5 min to complete. The primary outcome measure is the number of pumps made on balloons that did not burst. In summary, the Circle Tracing Task, Go/No-Go Task, 1-back task, and Wisconsin Card Sorting Task were employed to assess key cold executive functions, encompassing ongoing and prepotent inhibitory control, working memory, and cognitive flexibility in sequence. Additionally, the Balloon Analogue Risk Task was utilized as a measure of hot executive function, specifically targeting risky decision-making.

### tRNS protocol

**Figure 1:**
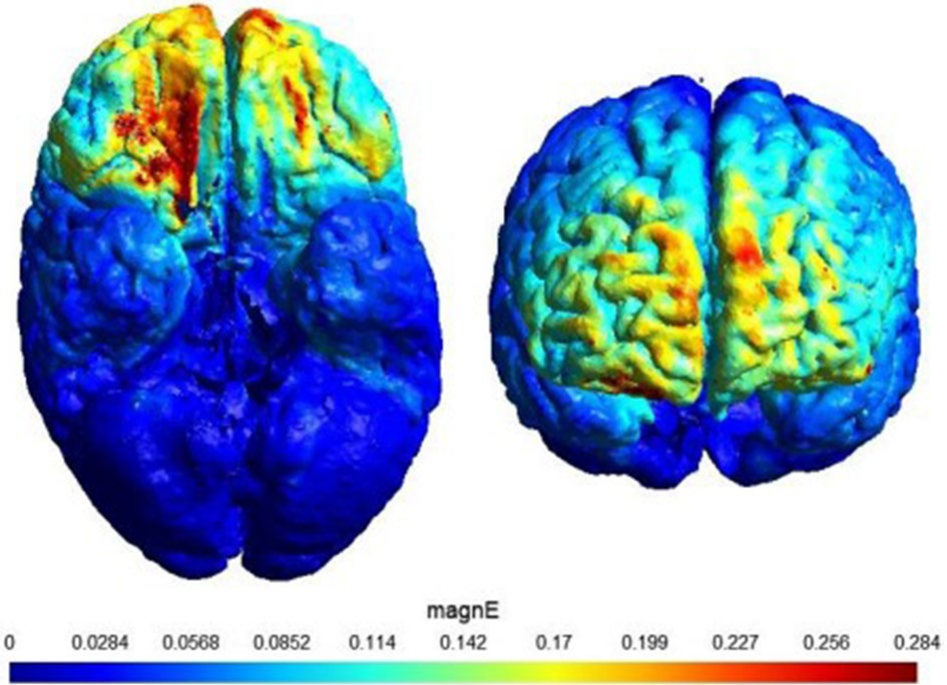
Distribution of electrical field calculated using SimNIBS in the frontal view (right) and inferior view (left). Two 5 × 5 cm electrodes were positioned over F3 and FP2 and the current intensity was set to 1 mA.

The Neurostim2 transcranial stimulator (Medinateb, Iran) was used to provide the tRNS protocol. High-frequency tRNS ranging from 100 to 640 Hz was delivered with an intensity of 1 mA (peak to peak), corresponds to a range between − 500 and 500 uA. The tRNS samples were generated using a uniform probability distribution, ensuring an equal likelihood of amplitudes between − 500 and 500 uA. The stimulation was applied through a pair of sponge electrodes, each measuring 5 × 5 cm, soaked in saline solution for optimal conductivity. This frequency and intensity have been previously employed in studies involving children with ADHD^34,45^. The electrodes were placed over the left dlPFC and the right vmPFC, and electrode placement was based on the 10–20 EEG international system (electrode positions with the center over F3, and FP2). The selection of these stimulation locations was based on their involvement in hot and cold executive functions, as suggested by previous studies^17,33,46^. Ramp-up and ramp-down durations were 30 s. The stimulation duration was 20 min. In the sham session, the current was only ramped up for 30 s to simulate the same sensation as in the real condition, and then ceased (Palm et al., 2013). The real and sham stimulation conditions were performed in two separate sessions with one week (± 24 h) interval. The illustration of modeling the flow of electrical current to examine the electric field distribution using SimNIBS is depicted in Fig. 1.

### Procedure

Prior to the experiment, the participants' parents provided written informed consent and assessed their children's ADHD symptoms using the SNAP-IV. The participants received the stimulation sessions in counterbalanced order, and were not aware of the stimulation type (real or sham) of each session. The stimulation duration was 20 min. After 5 min of stimulation, the participants performed the circle tracing task, Go/No-Go task, 1-back task, WSCT, and BART in counterbalanced order, which took approximately 15 min. At the end, the participants completed a side-effect checklist^47^, and were asked to guess which experimental condition they had received (real or sham).

### Data analysis

We employed the statistical software package SPSS version 23 for Windows to perform the data analysis. The Kolmogorov–Smirnov test was applied to evaluate normal distribution of the data. To examine the impact of tRNS on task performance and reported side effects under real and sham stimulation conditions in a within-subject design, we conducted repeated measures one-factorial analyses of variance (ANOVA) with the within-subject factor Stimulation (real and sham conditions). The dependent variables were the outcome measures of the tasks, as stated above, including circle tracing task (time index), Go/No-Go task (Go-accuracy and reaction time, No-Go accuracy), 1-Back task (1-Back accuracy and reaction time), WCST (perseveration error, cluster, and total time), and BART (adjusted value and successful pumps). Mauchly's Test of Sphericity was used to assess sphericity of data, and degrees of freedom were adjusted according to the Greenhouse–Geisser method when necessary. A Chi-square test was performed for assumed and real stimulation guesses for evaluation of blinding success. Additionally, session and task order were included as covariates in an additional ANCOVA. Bonferroni correction was applied to correction for multiple comparison. Statistical comparisons were rated significant at a p-value of less than 0.05.

## Results

With respect to the tRNS sessions, the ability of the participants to guess the type of stimulation was not better than chance (49% correct, χ2(1) = 1.37, *p* = 0.423). Furthermore, in both, real and sham stimulation conditions, they did not experience any side effects, such as pain, vertigo, burning, tingling, confusion, drowsiness, or nausea. Participants were able to complete the cognitive tasks properly after receiving initial instructions. Table 2 shows descriptive statistics of the task measures and the results of the respective one-way repeated measures ANOVAs.

**Table 2:** The mean and reaction time of task measures and ANOVA analysis.

Measures	M (SD)		ANOVA results			
	Real	Sham	df	F	*p*	ηp^2^
Circle tracing task						
Circle tracing index	56.305 (36.134)	45.397 (25.649)	1	6.045	.025	.262
Go/No-Go task						
Go accuracy	93.053 (6.230)	91.432 (5.047)	1	1.210	.287	.066
Go RT (s)	1.255 (.221)	1.306 (.206)	1	2.209	.156	.115
No-Go accuracy	97.861 (2.370)	95.937 (5.020)	1	4.638	.046	.214
1-Back task						
1-Back accuracy	73.777 (12.553)	68.611 (12.214)	1	6.480	.021	.276
1-Back RT (s)	2.022 (.521)	2.235 (.700)	1	1.816	.196	.096
Wisconsin card sorting task						
Total time (s)	287.744 (79.387)	368.289 (167.366)	1	5.368	.033	.240
Clusters	3.333 (.907)	3.111 (.758)	1	1.659	.215	.089
Perseveration error	11.222 (3.209)	12.055 (3.505)	1	.408	.531	.023
Speed accuracy ratio	7.820 (2.133)	10.307 (4.392)	1	6.541	.020	.278
Balloon analogue risk task						
Successful pumps	529.944 (287.267)	408.000 (160.863)	1	7.549	.014	.308

**Figure 2:**
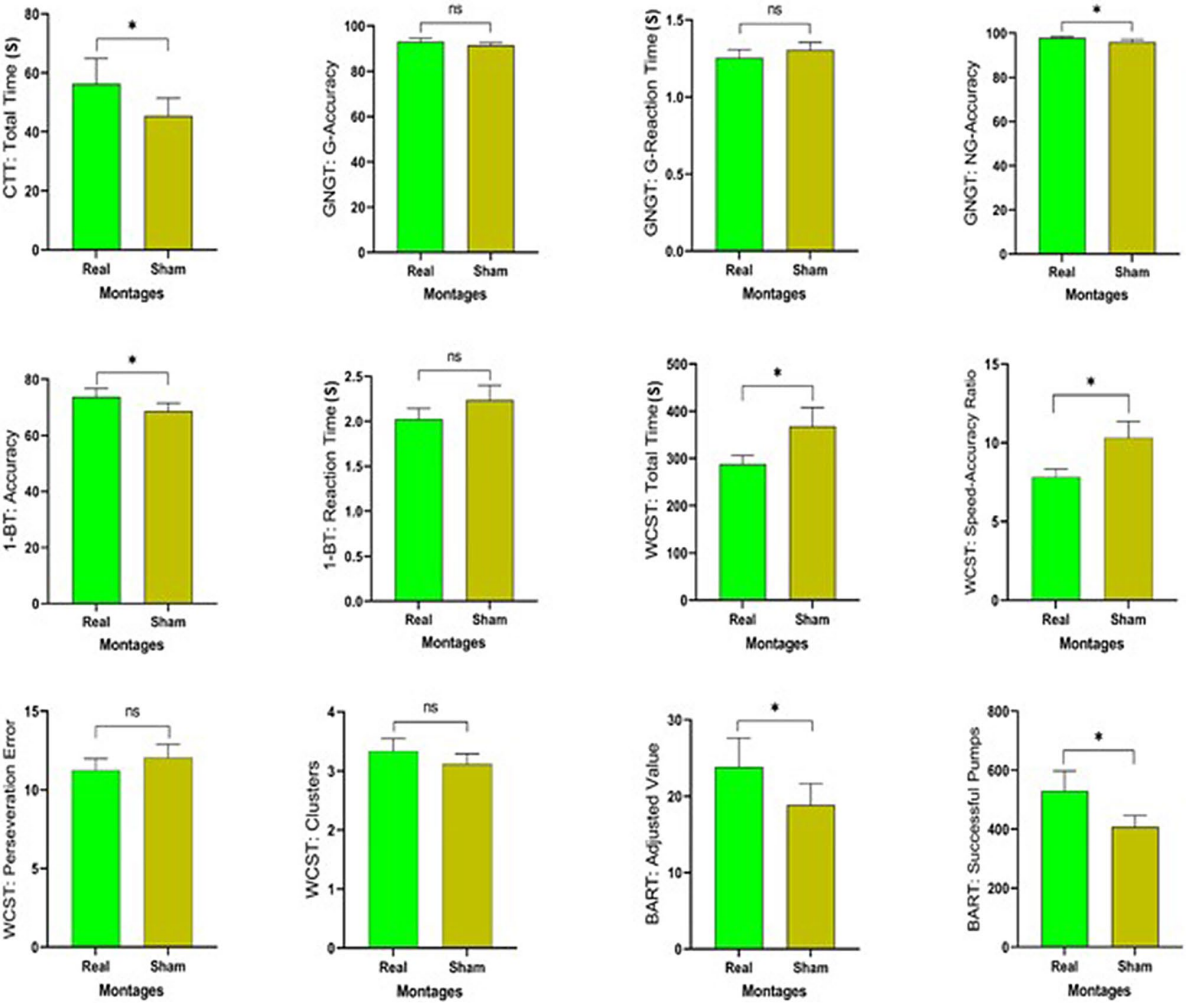
Shown are the effects of tRNS on the outcome measures. The vertical axis indicates the respective outcome measures of the tasks. The bars are showing the means, error bars represent standard error of means. The horizontal axes are showing stimulation conditions. *CCT* circle tracing task, *GNGT* Go/No-Go task, *1BT* 1-back task, *WCST* Wisconsin card sorting task, *BART* balloon analogue risk task.

Regarding the circle tracing total time, the ANOVA results indicate a significant difference between active and sham tRNS conditions, with a longer total time for the real stimulation, indicating larger ongoing inhibition during stimulation (MD (mean difference) = 10.908, *p* = 0.025). For the Go/No-Go task, the ANOVA results did not reveal any significant difference between real and sham stimulation for Go accuracy and Go reaction time. However, there was a significant main effect of stimulation for No-Go accuracy, the main outcome parameter of this task, with larger accuracy in the real tRNS compared to sham stimulation (MD = 1.924, *p* = 0.046), Fig. 2.

For the 1-Back task, there was a significant main effect of stimulation with respect to 1-Back accuracy, but not 1-Back reaction time. Comparison of the means showed higher accuracy for the active, compared to the sham stimulation (MD = 5.167, *p* = 0.021). For the WCST, a significant difference between real and sham tRNS conditions, with a longer total time in the sham stimulation condition, emerged (MD = 80.545, *p* = 0.033). However, no significant differences between conditions for clusters and perseveration error were observed. A significant difference between real and sham stimulation conditions for the speed accuracy ratio was revealed, with higher scores, and thus worse performance, in the sham condition (MD = 2.488, *p* = 0.020).

For the BART, significant differences were found between real and sham stimulation conditions regarding adjusted value and successful pumps, the main outcome measures of the task. Comparison of the means showed that participants scored higher in the active session for both, the adjusted value (MD = 4.947, *p* = 0.010), and successful pumps (MD = 121.944, *p* = 0.040) measures, compared to the sham condition. The significant results survive Bonferroni correction. No significant effects emerged for the co-variates of task and session order for any of the variables.

## Discussion

This study aimed to assess the impact of simultaneous stimulation of the dlPFC and vmPFC, using tRNS, on both cold and hot executive functions in children with ADHD. The results show an improvement of both, hot and cold executive functions during simultaneous upregulation of the vmPFC and the dlPFC through tRNS. This effect was shown for cold executive functions, including inhibitory control (measured by Circle Tracing and Go/No-Go tasks), working memory (measured with the 1-back task), but not for the crucial performance parameters of set shifting (measured through the Wisconsin Card Sorting test). Additionally, conservative decision-making, which is considered to indicate improved hot executive functions, was enhanced during stimulation. Overall, the simultaneous upregulation of the vmPFC and dlPFC through tRNS appears to have positive effects on both, cold and hot executive functions. This study is the first which applied tRNS simultaneously over the vmPFC and the dlPFC. Furthermore, this is the first tES study which simultaneously targets these areas with excitatory stimulation. In the following, we discuss our results based on the causal role of these areas for respective executive functions in detail, based on earlier tES studies. Because of the combined stimulation of both areas, our study did not specifically focus on the isolated role of these areas.

### Improved inhibitory control

Two aspects of inhibitory control, ongoing and prepotent inhibition, were measured in the present study through circle tracing and Go/No-Go tasks respectively. For prepotent inhibition, a longer index signifies enhanced ongoing inhibition during tRNS compared to the sham condition. Based on our literature review, to date, only one study has employed the Circle Tracing Task during brain stimulation in children with ADHD. This study reported improved ongoing inhibition during anodal right dlPFC/cathodal left vmPFC stimulation^35^. While the current study differs in both the stimulation parameters and target structures, the observed beneficial effects of both right and left dlPFC stimulation on ongoing inhibition suggest a shared underlying neural mechanism.

For prepotent inhibition, the results of the present study showed improved No-Go accuracy during stimulation compared to sham tRNS. Several tDCS studies in children with ADHD showed that anodal left dlPFC stimulation with the reference electrode over the right vmPFC^48,49^, right primary motor area^50^ or right dlPFC^35,36,51^ found no effect of stimulation on No-Go accuracy. This makes it improbable that right vmPFC stimulation in the present study caused these results. Soltaninejad et al.^52^ found improved No-Go accuracy during anodal right vmPFC/cathodal left dlPFC tDCS, indicating a role of the right vmPFC in response inhibition in children with ADHD. This is substantiated by improved No-Go accuracy during anodal right vmPFC/cathodal left dlPFC tDCS in children with ADHD^53^. In another tDCS study, anodal right dlPFC stimulation with an extracranial return electrode improved No-Go accuracy only in mildly affected ADHD children^35^. A cognitive modeling study on the data of that study found that stimulation reduced the tendency to action and raised the tendency to inhibition^54^. Given the role of the right dlPFC in inhibitory control based on these studies, improved inhibitory control during cathodal left dlPFC might be explained in the light of interhemispheric inhibition. Therefore, the role of the right dlPFC in inhibitory control could be boosted through direct anodal right dlPFC stimulation and indirect cathodal left dlPFC stimulation. In line with this assumption, bilateral anodal dlPFC stimulation, with two channels, with extracranial return electrodes, had no effect on No-Go accuracy in children with ADHD^36^. Similarly, bilateral tRNS over the left and right dlPFC in healthy participants showed no improvement of No-Go accuracy^55^. In sum, based on previous ADHD studies, improved prepotent inhibition has been described during upregulation of the right dlPFC, coupled with an extracranial reference electrode^35,56^. This effect vanished when simultaneously stimulation with identical polarity was applied to the left dlPFC^36^, indicating transcallosal inhibition. Moreover, improved prepotent inhibition has been described during upregulation of the right vmPFC either combined with an extracranial return electrode^57^, or left dorsolateral prefrontal cathodal stimulation^52,53^. Upregulation of the left dlPFC had no effect on prepotent inhibition^48,50–53^, but downregulation of it coupled with upregulation of the right vmPFC improves prepotent inhibition^52,53^. Although in the current study we did not aim to define the isolated roles of the dlPFC and vmPFC in prepotent inhibition, this pattern of results is compatible with a beneficial impact of an excitability enhancement of the right prefrontal cortex, and thus in our study the right vmPFC, on prepotent inhibition. Furthermore, we cannot attribute the enhanced prepotent inhibition to the upregulation of the left dlPFC, even though it was concurrently coupled with upregulation of the dlPFC.

### Improved working memory

The results show increased accuracy of N-back test performance, but not response time, during tRNS compared to sham condition. Accordingly, a meta-analysis of 16 tDCS studies show improved accuracy, but not reaction time, of working memory performance during anodal left dlPFC stimulation with a supraorbital return electrode in different psychopathological conditions^58^. This positive impact of anodal left dlPFC/cathodal right vmPFC stimulation on working memory performance has been described also in children with ADHD^34,59^. An ADHD study compared anodal left dlPFC/cathodal right vmPFC stimulation with tRNS over the right and left dlPFC, and found a larger tRNS effect on working memory accuracy, measured by the backward digit span test, compared with tDCS^37^. Another study compared anodal left dlPFC/cathodal right vmPFC stimulation with tRNS with the same electrode placement in healthy adults and found that tRNS induced more pronounced and consistent enhancements of working memory accuracy when compared to both, tDCS and sham stimulation^60^. In sum, left dlPFC stimulation, via either anodal tDCS or tRNS, improved working memory performance, and thus it is plausible that in the present study tRNS over the left dlPFC was causing these effects.

### Relatively improved cognitive flexibility

The WCST results showed a trend, but not significant effect of tRNS on the number of correct clusters and perseveration errors, as the main outcome measures of this test. However, response time decreased significantly during active stimulation compared to the sham condition. Given a similar outcome for the reaction time/accuracy trade-off^61^, this reduction could be interpreted as performance improvement, as shown by the lower speed-accuracy ratio during real, as compared to sham tRNS. It is worth mentioning that cognitive flexibility is relatively intact in individuals with ADHD^62^, thus this minor effect might be related to a ceiling effect. However, a tDCS study in children with ADHD showed improved cognitive flexibility in an experiment with two real stimulation conditions compared to sham: anodal left dlPFC/cathodal right vmPFC, and the reversed electrode arrangement. This study found moreover a larger impact of anodal left dlPFC/cathodal right vmPFC stimulation on performance. Another experiment of this study did not report improvement in cognitive flexibility during anodal right cathodal left dlPFC stimulation and an reversed order electrode placement compared to sham condition^53^. A bilateral anodal dlPFC stimulation study showed no alteration of WCST measures during real, as compared to sham stimulation^36^. Given different stimulation modalities and target areas, we cannot make a clear conclusion about the role of the dlPFC and vmPFC in cognitive flexibility based on these studies. In sum, the improved cognitive flexibility performance during anodal dlPFC/cathodal vmPFC stimulation, and the reversed electrode placement, as compared to sham tDCS, and a relatively minor effect of simultaneous stimulation of both, vmPFC and dlPFC through tRNS in this study suggests a possible see-saw effect between these areas in cognitive flexibility.

### Improved decision making

The results showed more conservative decision making, based on the balloon analogue risky decision-making task during the real stimulation condition, as compared to the sham condition. Reduced risky decision making has been described earlier during anodal right vmPFC/cathodal left dlPFC tDCS in children with ADHD^17^. Moreover, several tDCS studies described more conservative decision making during right/left dlPFC stimulation, independent of stimulation polarity^63–67^. Thus, the role of both, the vmPFC and dlPFC in decision making has been described earlier for tDCS. Earlier tDCS studies consider different roles of these areas for risky decision making. The vmPFC is suggested to play a crucial role in value-based decision making by integrating emotional and cognitive information to guide behavior^68^, whereas the dlPFC is suggested to be involved in memorizing and analyzing choices, and inhibit selection of irrelevant choices^69^. The present study confirmed the involvement of these areas by simultaneous stimulation of these areas. However, based on our stimulation protocol, this study does not allow to discern between the contribution of both areas.

### Limitations and future directions

This study has certain limitations, which should be taken into account. First, regarding the experimental design, our exploratory study had a one-session intervention approach and collected online surrogate markers, which limit the ability to draw strong conclusions about the clinical relevance of the intervention. To gain more information about its clinical relevance, future studies should incorporate multi-session stimulation and evaluate its impact on clinical and behavioral symptoms. Second, the sample size of this pilot study was moderate and the design was single-blind. For future studies, we suggest a larger sample size and a double-blind design to improve the rigor of the design. Third, the washout period of medication was 12 h before each stimulation session, and thus largely less than five half-lives of fluoxetine (1–3 days) and methylphenidate (2–3 h). Forth, another limitation of our study is the potential for unintended stimulation of regions beyond the targeted left dlPFC and right vmPFC, such as the right dlPFC, due to the spatial resolution constraints inherent in tES techniques. Additionally, future studies should include physiological markers to understand the underlying mechanisms of the observed cognitive effects, and titrate the intervention dosage according to intensity and duration for personalized therapeutic approaches.

### Conclusion

Hot and cold executive functions refer to two distinct types of cognitive processes. Hot executive functions refer to motivational processing, whereas cold executive functions require logical reasoning and goal-oriented behavior, such as working memory, inhibitory control and cognitive flexibility. In a broader concept, Nejati^70^ developed a matrix of higher cognitive functions based on types of information and processing styles. This matrix considers four classes for higher cognitive functions: Hot cognition, cold cognition, warm cognition, and cool cognition. Hot cognition refers to intuitive processing of emotional stimuli. Cold cognition refers to analytic processing of non-emotional stimuli, including cognitive flexibility, working memory, and inhibitory control. Warm cognition indicates intuitive processing of non-emotional stimuli, such as risky decision making and delay discounting. Cool cognition refers to analytic processing of emotional stimuli, such as emotion regulation and cognitive biases. At the neural level, the vmPFC is mainly associated with intuitive or emotional processing, while the dlPFC is more commonly associated with analytical or logical processing^71^. The findings of this study suggest that when the vmPFC and the dlPFC are simultaneously upregulated through tRNS, the main domains of both cold and hot executive functions are improved, including inhibitory control, set shifting, working memory, and decision-making. Earlier tDCS studies which target these areas discussed the isolated role of these areas^18,49,72–75^ or took the role of the vmPFC not specifically into consideration, because in many studies this area was downregulated by the cathodal return electrode. The former studies assumed a "see-saw" relationship between the dlPFC and the vmPFC, where activation of one region would result in inhibition of the other. Based on this assumption, the tDCS studies use anodal and cathodal electrodes to upregulate and downregulate these areas for modulation. However, in this study, we found that upregulation of both regions simultaneously led to an improvement of both, hot and cold executive functions.

## Data Availability

The datasets generated during the current study are available from the corresponding author on reasonable request.
